# Supplementary material for: HIVprotI: an integrated web based platform for prediction and design of HIV proteins inhibitors
Source: J Cheminform. 2018 Mar 9;10:12. doi: 10.1186/s13321-018-0266-y (PMC5845081; doi:10.1186/s13321-018-0266-y)
Supplement: Supplementary file 2 — Additional file 2. Source code of HIVProtI web server. [file 13321_2018_266_MOESM2_ESM.zip › HIVprotI_Source-code/hivprotI/weka-3-6-6/doc/weka/classifiers/pmml/consumer/PMMLClassifier.html]

PMMLClassifier


---


|  |  |  |
| --- | --- | --- |
| **Overview** \| **Package** \| **Class** \| **Tree** \| **Deprecated** \| **Index** \| **Help** \| **Weka's home** | |  |
| **PREV CLASS**   **NEXT CLASS** | **FRAMES**    **NO FRAMES**     **All Classes** |  |
| SUMMARY: NESTED \| FIELD \| CONSTR \| METHOD | DETAIL: FIELD \| CONSTR \| METHOD |  |


---


## weka.classifiers.pmml.consumer Class PMMLClassifier

```
java.lang.Object
  weka.classifiers.Classifier
      weka.classifiers.pmml.consumer.PMMLClassifier
```

**All Implemented Interfaces:**: java.io.Serializable, java.lang.Cloneable, CapabilitiesHandler, OptionHandler, PMMLModel, RevisionHandler

**Direct Known Subclasses:**: GeneralRegression, NeuralNetwork, Regression

---

``` public abstract class PMMLClassifier extends Classifier implements java.io.Serializable, PMMLModel ```

Abstract base class for all PMML classifiers.

**Version:**
:   $Revision: 5562 $

**Author:**
:   Mark Hall (mhall{[at]}pentaho{[dot]}com)

**See Also:**: Serialized Form

---

| **Method Summary** | |
| --- | --- |
| `void` | `buildClassifier(Instances data)`             Throw an exception - PMML models are pre-built. |
| `void` | `done()`             Signal that a scoring run has been completed. |
| `java.lang.String` | `getCreatorApplication()`             Get the name of the application that created this model |
| `Instances` | `getDataDictionary()`             Get the data dictionary. |
| `java.lang.String` | `getFieldsMappingString()`             Get a textual description of the mapping between mining schema fields and incoming data fields. |
| `Logger` | `getLog()`             Get the logger. |
| `MiningSchema` | `getMiningSchema()`             Get the mining schema for this model. |
| `java.lang.String` | `getPMMLVersion()`             Get the PMML version used for this model. |
| `void` | `mapToMiningSchema(Instances dataSet)`             Map mining schema to incoming instances. |
| `void` | `setCreatorApplication(org.w3c.dom.Document doc)`             Set the name of the application (if specified) that created this model |
| `void` | `setLog(Logger log)`             Set a logger to use. |
| `void` | `setPMMLVersion(org.w3c.dom.Document doc)`             Set the version of PMML used for this model. |

**Methods inherited from class weka.classifiers.Classifier**

`classifyInstance, debugTipText, distributionForInstance, forName, getCapabilities, getDebug, getOptions, getRevision, listOptions, makeCopies, makeCopy, setDebug, setOptions`

**Methods inherited from class java.lang.Object**

`equals, getClass, hashCode, notify, notifyAll, toString, wait, wait, wait`

**Method Detail**

### setPMMLVersion

```
public void setPMMLVersion(org.w3c.dom.Document doc)
```

:   Set the version of PMML used for this model.

    :   **Specified by:**: `setPMMLVersion` in interface `PMMLModel`
    :   **Parameters:**: `doc` - the Document encapsulating the pmml

---


### setCreatorApplication

```
public void setCreatorApplication(org.w3c.dom.Document doc)
```

:   Set the name of the application (if specified) that created this
    model

    :   **Specified by:**: `setCreatorApplication` in interface `PMMLModel`
    :   **Parameters:**: `doc` - the Document encapsulating the pmml

---


### getDataDictionary

```
public Instances getDataDictionary()
```

:   Get the data dictionary.

    :   **Returns:**: the data dictionary

---


### getMiningSchema

```
public MiningSchema getMiningSchema()
```

:   Get the mining schema for this model.

    :   **Specified by:**: `getMiningSchema` in interface `PMMLModel`
    :   **Returns:**: the mining schema

---


### getPMMLVersion

```
public java.lang.String getPMMLVersion()
```

:   Get the PMML version used for this model.

    :   **Specified by:**: `getPMMLVersion` in interface `PMMLModel`
    :   **Returns:**: the PMML version

---


### getCreatorApplication

```
public java.lang.String getCreatorApplication()
```

:   Get the name of the application that created this model

    :   **Specified by:**: `getCreatorApplication` in interface `PMMLModel`
    :   **Returns:**: the name of the creating application or null if not specified in the pmml.

---


### setLog

```
public void setLog(Logger log)
```

:   Set a logger to use.

    :   **Specified by:**: `setLog` in interface `PMMLModel`
    :   **Parameters:**: `log` - the logger to use

---


### getLog

```
public Logger getLog()
```

:   Get the logger.

    :   **Specified by:**: `getLog` in interface `PMMLModel`
    :   **Returns:**: the logger (or null if none is being used)

---


### buildClassifier

```
public void buildClassifier(Instances data)
                     throws java.lang.Exception
```

:   Throw an exception - PMML models are pre-built.

    :   **Specified by:**: `buildClassifier` in class `Classifier`
    :   **Parameters:**: `data` - the Instances to learn from **Throws:**: `java.lang.Exception` - if something goes wrong

---


### done

```
public void done()
```

:   Signal that a scoring run has been completed. Resets
    the initialized state to false so that a subsequent
    scoring run will trigger the mapping of the mining
    schema to incoming instances. If not called after a
    scoring run, then the classifier will assume that
    the current mapping is still valid.

---


### mapToMiningSchema

```
public void mapToMiningSchema(Instances dataSet)
                       throws java.lang.Exception
```

:   Map mining schema to incoming instances.

    :   **Parameters:**: `dataSet` - the structure of the incoming Instances **Throws:**: `java.lang.Exception` - if something goes wrong

---


### getFieldsMappingString

```
public java.lang.String getFieldsMappingString()
```

:   Get a textual description of the mapping between mining schema
    fields and incoming data fields.

    :   **Returns:**: a description of the fields mapping as a String or null if no mapping has been constructed yet.


---


|  |  |  |
| --- | --- | --- |
| **Overview** \| **Package** \| **Class** \| **Tree** \| **Deprecated** \| **Index** \| **Help** \| **Weka's home** | |  |
| **PREV CLASS**   **NEXT CLASS** | **FRAMES**    **NO FRAMES**     **All Classes** |  |
| SUMMARY: NESTED \| FIELD \| CONSTR \| METHOD | DETAIL: FIELD \| CONSTR \| METHOD |  |


---
